# Guidelines for hospital nephrology assistance from the Brazilian Society of Nephrology (BSN)

**DOI:** 10.1590/2175-8239-JBN-2024-0239en

**Published:** 2025-05-30

**Authors:** Mauricio Younes-Ibrahim, Eduardo Rocha, Thiago Reis, Vinícius Sardão Colares, Emerson Quintino de Lima, Lucia da Conceição Andrade, Eduardo Cantoni Rosa, Helen Siqueira Cardoso, Fernando Thomé, Daniela Ponce, José H. Rocco Suassuna, Luis Yu

**Affiliations:** 1 Sociedade Brasileira de Nefrologia, Departamento de Injúria Renal Aguda, São Paulo, SP, Brazil.; 2 Universidade Estadual de São Paulo, Departamento de Clínica Medica, Serviço de Nefrologia, São Paulo, SP, Brazil.

**Keywords:** Brazilian Society of Nephrology, Practice Guideline, Hospital Care, Nephrology, Acute Kidney Injury, Renal Dialysis

## Abstract

The AKI Department of the Brazilian Society of Nephrology (BSN) has prepared a hospital nephrology assistance guide, which encompasses the aspects involved in the nephrologist's role in caring for patients with kidney diseases within the hospital setting. The guide addresses the following main topics: 1) the role of the nephrologist in hospital care; 2) non-dialysis kidney support therapy; 3) technical standards for hospital dialysis care; and 4) outpatient follow-up of patients with acute kidney injury/disease. It provides a detailed description of the nephrologists' main responsibilities, their role in both non-dialysis and dialysis hospital care, as well as describing all available dialysis methods, the required infrastructure, human resources, and records of these procedures. The guide concludes with recommendations for the outpatient follow-up of nephrological patients after hospital discharge. The primary purpose of this BSN guide is to provide support for a better medical and multidisciplinary assistance for nephrologists and other professionals involved in the hospital patient’s nephrology care.

## The Role of the Nephrologist in Hospital Care

Nephrology is a medical specialty formally established in the 1960s and recognized by the Brazilian Medical Association (AMB). To be considered a nephrology specialist, a physician must obtain a board certification issued by the Ministry of Education and/or the Brazilian Society of Nephrology/AMB, duly registered with the respective Regional Council of Medicine.

The nephrologist’s role within the hospital setting is wide-ranging, and the competencies of the specialty include:

1. Consulting on projects for the installation and use of equipment specifically related to the specialty
2. Definition of care protocols for nephrology
3. Coordination of the activities of the multi­disciplinary team involved in nephrology care
4. Availability to provide nephrology consultations in support of other physicians within the hospital’s clinical staff
5. Continuous updating on new knowledge and emerging technologies in nephrology, to be applied in the hospital setting
6. Nephrologist participation in quality and safety committees aimed at establishing and disseminating initiatives for the prevention and early recognition of kidney function deterioration
7. The role of the nephrologist as an educator and disseminator of best practices for the management of these patients, considering that many patients hospitalized with acute kidney injury (aki) will be cared for by non-specialist physicians
8. Responsibility for the nephrological care and treatment of hospitalized patients, including:
  Identification of patients at risk of developing renal dysfunction;
  Preventive and protective measures for kidney diseases;
  Individualized adjustment of drug prescri­ptions to the corresponding renal function, aiming to prevent nephrotoxicity events;
  Prevention and monitoring of complications from in-hospital procedures with potential impacts on renal function;
  Definition and use of relevant complementary methods to assist and refine the diagnosis of kidney diseases;
  Indication oF renal replacement therapy (rrt) methods;
  Prescription and supervision of rrt procedures;
  Provision of vascular and peritoneal accesses required for rrt;
  Management of continuous r therapies in critically ill patients, in conjunction with the medical teams of closed units;
  Interaction with multidisci­plinary teams to define shared therapeutic strategies;
  Clinical activities related to the preparation and kidney transplants procedures;
  Preparation and referral for nephrology follow-up and/or rrt orientation after hospital discharge;
  Careful identification of selected cases for palliative care.
9. The presence of a nephrologist in the hospital setting is essential for prescribing and monitoring nephrological procedures. the use of telemedicine for remote hospital nephrology activities only applies to interdisciplinary teleconsultation with the medical team providing care to the patient, and it is not applicable to inpatient teleconsultation or for the prescription of renal replacement therapy.
10. Labor relations (public and private nephrology services)
  The brazilian society of nephrology recommends that each hospital should have an identified, permanent team of nephrologists – either in-house or contracted – working integratively with the clinical staff and exercising completely the responsibilities of the specialty. the multiple competencies outlined above require specific agreements on the respective remunerations.

## Non-Dialysis Kidney Support Therapy

The nephrologist, a mandatory member of the hospital clinical staff, is the professional qualified to provide care for patients with renal dysfunction, hydroelectrolytic disturbances, and acid-base disorders. This specialist is also skilled at devising AKI prevention strategies, interacting with the multidisciplinary team, and drafting protocols to standardize the care that covers most cases, while maintaining the flexibility to individualize care in specific situations. In this sense, the participation of nephrologists in quality and safety committees aimed at developing strategies for the prevention of nephrotoxicity, performing preoperative clinical assessment of patients with impaired renal function, or addressing electrolyte and acid-base disturbances is essential^1^.

Hospitalized patients with AKI will also be managed by non-nephrologist physicians. For this reason, the role of the nephrologist as an educator and disseminator of best practices for the management of these patients is crucial. Non-dialysis support strategies for AKI are similar to those used in its prevention^1^.

### Nephrologists should Operate on Several Fronts to provide non-dialysis Treatment in The Hospital Setting, Including

1. Working alongside hospital administrators and as coordinator of the local nephrology service;
2. Commitment to supporting infrastructure^1^;
3. Engagement with leadership across various units to align care practices, develop and oversee quality assessments, and foster a culture of success linked to nephrology-driven initiatives^1, 2, 3^;
4. Support from hospital leadership is essential for this action^1,2^.

### Participation in Quality and Safety Committees

Although some cases of AKI are potentially preventable, this condition has a high impact on hospital costs and the survival of hospitalized patients. Thus, the development of AKI may be considered a quality indicator of hospital care. The inclusion of nephrologists in quality and safety committees could help establish and disseminate initiatives for the prevention and early recognition of renal dysfunction^1,7,8^.

### Preoperative Assessment

Patients with chronic kidney disease (CKD) are at increased risk of perioperative complications^9^. A nephrology consultation is recommended during the preoperative assessment of these patients and of those at high risk for postoperative renal dysfunction. Follow-up of these patients is crucial for early intervention in modifiable risk factors and monitoring of non-modifiable ones^10^. For patients already on dialysis support, nephrology care is mandatory in order to determine the optimal timing for dialysis prescription in the pre- and postoperative periods. Patients without CKD but at high risk of developing renal impairment during surgery – such as elderly patients with multiple comorbidities, those on multiple medications, critically ill patients, and individuals with severe congestive heart failure or liver disease – should also be monitored by the nephrology team^10^.

### Care of Chronic Kidney Disease Patients

Patients hospitalized with CKD, including non-dialysis, dialysis, and kidney transplant patients, should be followed by a nephrologist so as to identify and correct drug interactions, adjust medications according to the degree of kidney dysfunction, avoid nephrotoxic drugs, and establish measures for nephrotoxicity prevention and the management of immunosuppressive drugs^10^. These activities may be carried out in conjunction with the clinical pharmacist, nutritionist, and attending physician to establish the most appropriate diet, volume management, correction of electrolyte and acid-base disturbances, manage vascular access, and optimize dialysis support.

### Care of Patients with AKI

Many patients hospitalized with AKI will be under the care of non-specialist physicians, reinforcing the relevance of the nephrologist as an educator and disseminator of best practices for the management of these patients^1^. In addition to the activities already described, their responsibilities include the early recognition of renal dysfunction, the development of strategies to prevent nephrotoxicity, blood volume control, the identification and correction of electrolyte and acid-base disorders, as well as drug adjustment to the degree of renal dysfunction.

The nephrologist’s role in the non-dialysis treatment of AKI patients also involves adequate control of the following clinical manifestations:

#### Hemodynamics and Fluids

1. In case of hypovolemia, administer appropriate hydration solutions;
2. The initial resuscitation target, particularly in patients with sepsis, is to maintain mean arterial pressure (map) above 65 mmhg. in patients with preexisting hypertension, higher targets may be considered^11, 12, 13, 14, 15, 16;^
3. Vasopressors may be needed to achieve pam targets. norepinephrine is the most commonly used vasopressor for patients with sepsis-related aki. in patients receiving norepinephrine who have difficulty achieving the target map, vasopressin may be added^13,17^;
4. Monitor intra-abdominal pressure (iap) in selected cases. renal perfusion depends on the difference between inflow pressure (map) and outflow pressure (central venous pressure – cvp). elevated cvp and renal venous congestion are associated with aki in patients with heart failure and abominal compartment syndrome. in the latter case, iap may exceed cvp, and renal perfusion pressure becomes the difference between map and iap^13,17^;
5. Avoid unnecessary volume expansion by controlling the administration of maintenance fluids and excessive dilution of medications, in addition to discouraging excessive fluid intake, especially in oliguric patients^18, 19, 20, 21, 22^;
6. Strict control of urine output, especially diuresis flow, is necessary^23^;

#### Use of Diuretics

1. Administer furosemide to treat hypervolemia and hyperkalemia. other diuretics (thiazides, acetazolamide, spironolactone, amiloride) may be combined to achieve dual or even triple blockade of the nephron segments^24^;
2. Do not delay the decision to initiate dialysis, especially when there is no response to diuretic administration; even if there is some response to diuretics, dialysis should be considered in the event of worsening renal function^25^;
3. The “furosemide stress test” (bolus infusion of 1 mg/kg of furosemide in euvolemic or hypervolemic patients, without prior diuretic use, or 1.5 mg/kg in patients previously exposed to diuretics) could be used to identify patients at greater risk of aki progression and the need for dialysis. these are patients who do not respond with diuresis > 200 ml within 2 hours of diuretic infusion^26^;

#### Nutrition

1. An intake of 20–30 kcal/kg/day should be targeted at all stages of aki^27^;
2. Avoid restricting protein intake with the aim of preventing or delaying dialysis initiation^27,28^;
3. Administer 0.8–1.0 g/kg/day of protein in aki patients not requiring dialysis, and 1.0–1.5 g/kg/day in patients on dialysis, up to a maximum of 1.7 g/kg/day in those on continuous dialysis or in hypercatabolic subjects^28^;
4. Avoid episodes of hyperglycemia, trying to maintain blood glucose levels below 180 mg/dl. glycemic control is essential in the care of critically ill and surgical patients, as both hyperglycemia and hypoglycemia are related to increased mortality and the occurrence of aki. kdigo recommends maintaining blood glucose levels between 110 and 149 mg/dl, with close monitoring to prevent hypoglycemia^29,30^;
5. Attention should be paid to the intake or administration of diets with excessive sodium, potassium, and phosphorus content, as well as fluid restriction, when necessary;

#### Acidosis and Hyperkalemia

1. If blood ph is < 7.2, particularly when associated with a serum bicarbonate level of less than 15 meq/l, and no evidence of hypervolemia, sodium bicarbonate could be administered^31^;
2. Attention to other causes of metabolic acidosis not caused by renal dysfunction, such as exogenous intoxications, lactic acidosis, and diabetic ketoacidosis^31^;
3. When correcting metabolic acidosis, a decline in ionized calcium may occur. if calcium correction is necessary, it should be infused into a separate intravenous line, due to the incompatibility of calcium and bicarbonate solutions^31^;
4. Treatment of hyperkalemia – the main nephrological emergency – must be promptly instituted. it should include measures to protect the myocardial membrane (calcium gluconate), measures to redistribute extracellular potassium into the intracellular compartment (glucose/insulin solution, b2-adrenergic agonists, and sodium bicarbonate) and removing potassium from the body (furosemide, ion exchange resins and binders, and dialysis). hyperkalemia is an extremely serious condition that may lead to death; therefore, dialysis should be rapidly initiated when there is no response to clinical measures^32^;

#### Anemia

1. Hemoglobin levels < 8 mg/dl are a risk factor for aki, both in the preoperative and postoperative periods, for most surgical procedures. preoperative optimization of hemoglobin levels is recommended to avoid blood transfusions and postoperative decompensation^33^.

## Technical Standards for Inpatient Dialysis Care

The provisions of this guideline apply to Nephrology services that offer RRT, peritoneal dialysis, and other extracorporeal clearance methods – procedures considered highly complex and performed in a hospital setting^34^.

### Indications

Indications for RRT include those considered urgent or classic (acidosis, electrolyte disturbances, drug intoxication, fluid overload, and uremia) or those based on demand (nutrition, drugs, blood products) versus renal reserve. These are assessed through the evolution of nitrogen waste, volume overload, involvement of other organs, and the need for inflammatory mediator removal in the context of critically ill patients^35, 36, 37^.

The nephrologist should also consider the nature of the insult and the likelihood of recovery, the underlying disease, comorbidities, ethical considerations, and the risks of the procedure^38, 39, 40, 41, 42, 43, 44^.

### Extracorporeal Modalities/Methods

#### Description

Considering the mechanisms of solute exchange (diffusion, convection, and ultrafiltration), preferably used in the three preferred dialysis modalities (conventional, prolonged, and continuous), the different methods to be applied to hospitalized CKD patients (hemodialysis, hemofiltration, hemodiafiltration, and ultrafiltration) will be designated (Table 1)

**Table 1:** Methods: modalities, mechanisms, and characteristics

		Modalities		
		Conventional RRT (CHD)	Prolonged RRT (PHD)	Continuous RRT (CRRT)
Mechanisms of Solute Exchange	Diffusion	Hemodialysis	Hemodialysis	Hemodialysis
	Convection	–	–	Hemofiltration
	Diffusion/Convection	–	Hemodiafiltration	Hemodiafiltration
	Ultrafiltration	Ultrafiltration	Ultrafiltration	Ultrafiltration
Kinetics – Efficiency		High	Medium	Low
Drug/toxin clearance per unit time		Higher	Intermediate	Lower
Kinetics – Weekly effectiveness, slag removal/inflammation over the course of treatment		Lower	Intermediate	Higher
Hemodynamic instability		Higher	Intermediate	Lower
Increased ICP		Higher	Intermediate	Lower
Volume status control		Lower	Intermediate	Higher
Anticoagulation		Dispensable	Dispensable	Yes/eventually dispensable
Compatibility with other extracorporeal procedures		No	No	Yes
Patient transportation*		Yes	Yes	No
Cost		Lower	Intermediate	Higher

Abbreviations – ICP: intracranial pressure; CHD: conventional hemodialysis; PHD: prolonged hemodialysis; RRT: renal replacement therapy; CRRT: continuous renal replacement therapy. Note – *Patient mobilization (e.g. sitting up in an armchair) depends on vascular access site and hemodynamic stability; however, leaving the bed for interventions and examinations becomes easier with intermittent modalities.

In the conventional hemodialysis (CHD) modality, the use of solute exchange in inpatient dialysis is preferably by diffusive method, in a high-efficiency regimen, thus predisposing to greater osmotic and fluid balance fluctuations, and, consequently, greater hemodynamic instability and a potential increase in intracranial pressure (expansion of the intracellular compartment)^45,46^.

In prolonged or extended hemodialysis (PHD), osmotic and fluid balance fluctuations are intermediate, still subject to instability^47^.

In continuous renal replacement therapy (CRRT), fluid removal occurs more slowly, kinetics of solute exchange are low, and convective methods may be used in combination, allowing for greater stability and suitability for more severe patients – especially those prone to intracranial hypertension, dysnatremia, and hypercatabolism^48,49^.

Ultrafiltration is aimed at ensuring the patient’s volume balance and may be indicated alone or in association with other methods in the different modalities (Tables 1 and 2)^50^. The characteristics of extracorporeal RRT modalities in AKI are summarized in Table 1 ^51, 52, 53^.

**Table 2:** Operating parameters in dialytic modalities

	Conventional HD	Prolonged HD	Continuous RRT
Sector	Hemodialysis room, Inpatient Unit ICU, Step-down Unit	ICU, Step-down Unit	ICU
Method	Hemodialysis Ultrafiltration alone	Hemodialysis Hemodiafiltration Ultrafiltration alone	Hemodialysis Hemofiltration Hemodiafiltration
Access	AVF, temporary or permanent catheter	AVF, temporary or permanent catheter	Temporary or permanent catheter
Equipment	Hemodialysis machine	Hemodialysis or continuous therapy machines	Continuous therapy machine
Device	Biocompatible polymer membrane	Biocompatible polymer membrane	Biocompatible polymer membrane
Session time	up to 6 h	6 to 12 hours	24 h
Frequency	3 to 4x/week or daily	3 to 4x/week or daily	Continuous
Dialysate flow	300–800 ml/min	100–300 ml/min	Variable, usually 20–35 ml/Kg/h (including replacement solution when convection occurs)
Blood flow ml/min	200–400	100–250	100–200 ml
Effective ultrafiltration	0–4 l	0–4 l	Variable, according to demand
Anticoagulation	With or without heparin (unfractionated or lmwh)	With or without heparin (unfractionated or lmwh); regional citrate	With or without heparin (unfractionated or lmwh); regional citrate
Target KT/V (session/wk)	0.9 to 1.3/2.0 to 2.5	0.9–1.0/3.0–3.5	20–30 ml/kg/h*

Abbreviations – AVF: arteriovenous fistula; lmwh: low-molecular-weight heparin. Note – *The dose of continuous RRT includes the total effluent flow (dialysate solution, hemofiltration + ultrafiltration, including, in some equipment, compensatory filtration to the citrate or calcium infusion).

### Choice of Modality

The choice of dialysis modality is individualized, considering the clinical picture of each patient, as well as the availability of equipment and the staff’s training on the different methods. This decision should be made by the nephrologist, in agreement with the patient’s intensivist or attending physician (Table 1)^54^.

#### Peritoneal Dialysis

Peritoneal dialysis (PD) is a therapeutic option for the treatment of patients with AKI, which has regained prominence following the COVID-19 pandemic. Some studies have demonstrated its non-inferiority in terms of outcomes for AKI patients when compared to extracorporeal therapy^55^.

In selected cases, PD may be a more appropriate option - for example, when anticoagulation is contraindicated, in situations where vascular access is difficult, in hypercoagulable states, after cardiac surgery, in limited local resources, and in pediatrics. In addition, PD does not cause dialysis disequilibrium syndrome, gradually corrects imbalances, is more biocompatible, and is suitable for cardiorenal syndrome situations and neurocritical patients^56,57^. However, it is contraindicated in cases of pleuroperitoneal communication, severe respiratory and diaphragmatic failure, unresponsive life-threatening hyperkalemia, severe hypercatabolism, low peritoneal clearance, abdominal wall cellulitis, large hernias/adhesions/peritoneal fibrosis, adynamic ileus, recent abdominal surgery, or other confirmed or suspected abdominal abnormalities. In addition, it is not safe for the treatment of acute pulmonary edema.

There are different types of peritoneal dialysis, allowing for therapeutic flexibility: intermittent (with short dwell times, lasting up to 24 hours, repeated as needed); continuous equilibration (with longer dwell times, in continuous cycles); current or tidal (similar to intermittent, but with residual peritoneal volume maintaining equilibrium during the cycles); and high-volume (with elevated total daily volumes). The volume of each cycle depends on the patient’s size, ranging from 1 to 3 liters in adults and 10–50 ml/Kg in children. There is a wide variation in practices, depending on individual services.

The most appropriate dose for PD in the management of AKI patients remains poorly defined. This lack of a clear definition is due to the limited number of studies available for comparing treatment using different modalities. Although Kt/V is considered to be optimal, it is not practical. In practice, adequacy is analyzed by clinical signs and fluid balance, normalization of potassium levels, and improvement in the acid-base balance.

### Technical Regulations

1. In order to provide optimal dialysis care for hospitalized patients, it is recommended that all responsible parties, including hospitals and nephrology services, comply with the regulatory guidelines set forth in this guide;
2. It is recommended that hospitals provide bedside nephrology support and renal replacement therapies for patients who require RRT and are unable to be removed to outpatient dialysis units, either in or out of hospital. Furthermore, hospitals must ensure optimal physical infrastructure conditions in hospital units (ICU or general wards) to perform RRT^58^;
3. The Nephrology service is responsible for indicating, installing, monitoring, and terminating dialysis treatment for inpatients, in accordance with protocols and standard operating procedures pre-established at the hospital^58^;
4. The co-responsible parties are required to provide the equipment, supplies, and medication necessary for the treatment of RRT, in accordance with the established contract.

### Multidisciplinary Team

The Nephrology service that provides dialysis care should be staffed by legally qualified professionals who meet the specific competencies for the processes to which they have been assigned. It must be supervised by a nephrologist designated as the technical supervisor (TS) and a nursing TS, both certified in Nephrology by their respective professional councils or specialty societies. They shall assume technical responsibility for the healthcare service before the sanitary authority, in accordance with current legislation.

These professionals should also design, implement, and monitor the established operating procedures and protocols, alongside the other medical and non-medical teams within the Nephrology Service and the hospital. In addition, they will be responsible for the staff roster and should attend hospital meetings related to patient safety, infection control, and water treatment.

The care process should also include other professionals, such as nephrologists, intensivists, and hospitalists; nephrology nurses; nursing technicians trained in the different RRT methods; vascular and general surgeons; pharmacists; and equipment technicians, each with specific responsibilities, namely:

− Nephrologists: in-person patient assessment; indication for treatment upon demand by the hospitalist or intensivist; prescription of RRT; prevention of procedure-related complications; insertion or request for vascular access; alteration of dialysis parameters when necessary (flow rates, electrolyte concentrations in solutions, adjustment of anticoagulation, change in modality or methods). It is recommended that the nephrologist serving as TS determine, in each context, the need for nephrologist’s presence or supervision during the delivery of intermittent therapies, as well as the appropriate workload the nephrologist can effectively manage^54,59^.
− Pediatrician: it is recommended that RRT in pediatric patients be conducted by a pediatric nephrologist. In facilities where there is no pediatric nephrologist available, RRT or bedside peritoneal dialysis may eventually be accompanied by an adult nephrologist and a pediatrician, both qualified to manage clinical complications.
− Hospitalists or intensivists: management of clinical complications; occasional insertion of vascular access and assessment of its correct position; adjustment of ultrafiltration intensity, as needed and in agreement with the nephrologist.
− Vascular and General Surgeon: insertion of vascular or peritoneal access.
− Nephrology nurse: installation and completion of continuous therapies; temporary withdrawal of treatment due to complications; vascular access care, including tractioning or changing vascular access routes when necessary, and arteriovenous fistula puncture when indicated.
− ICU nurses: monitoring of procedures and related complications, especially continuous therapies and treatment completion. When qualified in Nephrology, they can replace or work alongside Nephrology nurses. The puncture of arteriovenous fistulas or grafts is prohibited for nurses who are not trained for this purpose.
− Nephrology Nursing Technician: installation of conventional and prolonged HD; monitoring of bedside procedures; installation of CRRT under the supervision of a nurse; arteriovenous fistula puncture, when trained and experienced to do so, under the guidance and instructions of the nurse.
− Pharmacist: monitoring prescribed drug dosages, adjustments according to kidney function and ongoing treatment, supervision of solution preparation for CRRT in centers where in-house preparation is available.
− Equipment technician: preventive or corrective maintenance of RRT and water purification equipment.

### Infrastructure – Hospital Facilities

The hospital must provide, at the bedside, in the ward, intensive care, and step-down units: an adapted potable water outlet; a wastewater outlet for discharging effluent; and an adapted electrical network for installing dialysis and osmosis equipment.

Dialysis machines, as well as reverse osmosis equipment and the supplies used, must be duly registered with the Ministry of Health and approved by Anvisa (the National Health Surveillance Agency). Their operation must follow the manufacturer’s recommendations.

In addition, emergency care materials and equipment must be readily available and in perfect working condition, close to the dialysis sites.

The hospital should also provide a designated area for storing, cleaning, and disinfecting equipment and materials, as well as for the disposal of needles, lines, filters, pressure transducers and leftover solutions, which must not be reused. This area must remain clean, well-ventilated, protected from light, heat, and humidity. It should be equipped with a potable water outlet, a drainage point, an electrical outlet for equipment, and a sink for hand sanitizing^54,58^.

### Operating Parameters in Therapies

The Nephrology Service should preferably offer different dialysis modalities and methods that could be tailored to the various clinical scenarios presented by patients with AKI and those with CKD stage V. It is recommended that extracorporeal modalities and their respective methods (techniques), as well as PD, be made available by the hospital’s Nephrology Service.

Table 2 presents the operating parameters used in the modalities and methods most commonly employed in the management of AKI patients^60,61^.

The use of less common methods, such as therapeutic plasmapheresis, hemoperfusion, and artificial liver support, may also fall under the responsibility of the Nephrology team. These are illustrated in Table 3 ^62, 63, 64, 65^.

**Table 3:** Extracorporeal methods

	Hemoadsorption	Therapeutic plasma exchange	Molecular adsorbent recirculating system (MARS)	Single-pass albumin dialysis (SPAD)	Extracorporeal carbon dioxide removal (ECCO2R)
Target-removal	Middle molecules, drugs, toxins, endotoxins, bilirubin, and bile salts	Antibodies, toxins, immune complexes, blood figurative elements	Bilirubin and bile salts	Bilirubin and bile salts	Carbon dioxide
Sector	Hemodialysis room, Inpatient unit, ICU, Step-down Unit	Inpatient unit, ICU, Step-down Unit	ICU, Step-down Unit	Hemodialysis room, Inpatient unit, ICU, Step-down Unit	ICU
Method	Hemoperfusion	Hemofiltration	Hemodialysis, albumin hemoperfusion	Hemodialysis	Hemodialysis
Access	AVF, temporary, permanent catheter	Temporary, permanent catheter	Temporary, permanent catheter	Temporary, permanent catheter	Temporary, permanent catheter
Equipment (machines)	Hemodialysis, continuous therapy, specific for hemoperfusion; cartridges coupled with extracorporeal circuits	Hemodialysis, continuous therapy with plasmapheresis module	Specifically coupled to the continuous hemodiafiltration or hemodialysis machine	Continuous therapy	Specific for hemoperfusion, continuous therapy coupled to the oxygenating membrane
Devices	Cartridges with resins or microspheres	Membrane filters with biocompatible polymer	Membrane filters with biocompatible polymer, activated carbon circuit and ion exchange cartridge	Membrane filters with biocompatible polymer	CO2 removal membrane
Session time	2–24 h	2 h	6–8 h	7–8 h with albumin + 15–16 h hemodiafiltration	72 h
Frequency	Daily	Daily; every 48 hours	Daily	Daily	Daily
Length of treatment	72 hours or longer	Variable	72 hours or longer	72 hours or longer	72 hours or longer
Anticoagulation	Heparin, citrate or no anticoagulation	Heparin, citrate or no anticoagulation	Heparin, citrate or no anticoagulation	Heparin, citrate or no anticoagulation	Heparin or no anticoagulation
Specific solution	No	Albumin with saline solution or plasma	Solution with albumin	Hemodiafiltration solution with added albumin	No

### Solutions and Supplies

The solutions used in extracorporeal therapies enable the direct clearance of uremic toxins, in proportion to the blood flow in the conventional modality, and in direct relation to the dialysate flow in continuous therapies. Electrolyte clearance will depend on the gradient between serum concentration and the dialysate solution, and may be adjusted according to the concentrations of the electrolytes involved^66^.

In conventional HD, the dialysate is generated in real time. In CRRT, commercially available, ready-to-use solutions may be used, as may solutions prepared in-house, preferably by the hospital pharmacy. When the option is for the nursing team to prepare at the bedside, a safety protocol is indicated.

These solutions, together with citrate and calcium solutions used in CRRT, as well as those used in PD, should be stored in adequate locations for proper preservation.

### Vascular Access

Most hospitalized patients with AKI requiring RRT should have a temporary venous catheter inserted. Eventually, for stable patients, in whom a longer or definitive dialysis interval is expected, the placement of a long-term catheter may be indicated.

Central vein puncture should be guided by ultrasound, and the catheter’s position should be confirmed radiologically after insertion^67^.

Catheters generally have a diameter of 11 to 14 French, with a preference for 14 French catheters in the continuous modality, particularly when there is a tendency toward blood clotting.

According to KDIGO, catheters should be inserted following a preferred sequence of puncture sites: right internal jugular vein (16–20 cm), femoral vein – right or left – (24–30 cm), and left jugular vein (20–24 cm). The subclavian vein should only be used in exceptional circumstances. AVF access is reserved for patients on a chronic dialysis program who remain on conventional therapy^67^.

### Therapy Monitoring

Monitoring RRT in hospitalized patients, particularly in the conventional HD modality, involves observing potential hemodynamic instability, especially in patients with malnutrition, heart disease, those receiving hypotensive or vasoactive drugs (at low doses), and those transitioning from the continuous modality^68,69^.

It is recommended that potentially unstable patients undergo dialysis in a critical care environment, duly monitored in order to avoid hypotensive episodes.

Bedside hemodynamic monitoring, as well as volume status assessment using biomarkers, bioimpedance, echocardiography, and point-of-care ultrasound, allow for more accurate and safer removal rates^70,71^.

Biochemical control enables the monitoring of waste products normalization by the therapies and the consequent adjustments to the effluent doses. In addition, electrolytes and the acid-base profile should be monitored, with appropriate corrections made via the dialysate profile, according to the employed modality.

In the continuous modality, biochemical analyses are collected periodically, two to four times a day. These collections also include analyses related to the anticoagulation process employed, which is most often regional, using trisodium citrate. In such cases, control is performed by determining systemic and the post-filter circuit ionized calcium^72^.

### Complications

Complications of RRT methods are associated with the vascular access, the extracorporeal circuit, or the patient themselves.

During catheter insertion, bleeding, hematoma, failure to obtain access or primary nonfunction, arterial puncture, pneumothorax, hemothorax, air embolism, and cardiac arrhythmias may occur. The physician performing a venous access must be aware of these potential complications and be able to diagnose and manage them. They should also conduct a preliminary assessment of the patient, recognizing coagulopathies, the history of previous punctures, and the current clinical condition. The use of ultrasound to guide the puncture is an essential preventative measure^67^.

Following catheter insertion, insufficient arterial or venous flow, accidental disconnection, venous stenosis, thrombosis, bloodstream or ostial infections may occur. Preventing these complications requires strict aseptic protocols for both catheter insertion and care. Additionally, catheter use should be avoided for other functions. Inserting the correct catheter into the correct vessel prevents further complications.

Complications related to the extracorporeal circuit include air embolism, circuit clotting, hypothermia, equipment failure, and rupture of dialyzer fibers. Anticoagulation and equipment maintenance protocols must be strictly followed. Filtration fraction rates above 25% should be avoided, as should not deactivate the equipment alarms. Continuous monitoring of circuit pressures and other safety parameters is also mandatory.

Among the complications related to the patient, those resulting from anticoagulation include bleeding and heparin-induced thrombocytopenia, as well as electrolyte and acid-base disturbances due to the use of citrate.

Regional anticoagulation with citrate may cause: a) increased metabolism or failure in the clearance of citrate, leading to overload, characterized by metabolic alkalosis and hypernatremia. These can be corrected by reducing the dose, decreasing blood flow, increasing dialysis dose, or changing the composition of the dialysate or replacement solution; b) toxicity due to inadequate metabolism (associated severe liver failure, tissue hypoperfusion due to refractory shock), characterized by a reduction in systemic ionized calcium, an increase in the total calcium/ionized calcium ratio (> 2.5) – with special care to ensure both values are expressed in the same measurement unit (mg/dL or mmol/L) – and high anion gap metabolic acidosis. In the latter situation, citrate infusion should be discontinued.

Prolonged use of high doses in continuous methods may lead to electrolyte disturbances, with depletion of intracellular ions, hypophosphatemia, hypomagnesemia, or hypercalcemia secondary to bone calcium reabsorption due to prolonged immobility. Standardized solutions, containing fixed concentrations of electrolytes and bicarbonate, may render the adjustment of electrolytes, acid-base balance, and anticoagulation more complex.

Electrolyte disturbances could also be induced in the intermittent modality: rapid variations in potassium and sodium concentrations and in acid-base balance – which should be anticipated during procedure planning.

Hemodynamic instability is a frequent complication in RRT management. Hypotension occurs more frequently with intermittent methods, although it may also eventually occur with continuous methods. Rapid removal of fluids, in addition to the refilling capacity of the intravascular compartment, is the most common cause of instability. Other contributing causes include osmotic imbalances, myocardial stunning, vasodilation, and increased capillary permeability induced by immune activation in the extracorporeal system, occult bleeding, anemia, reactivation of septic conditions, mesenteric ischemia, respiratory acidosis, and excessive clearance of vasopressors^73^. Frequent assessment of the patient’s hemodynamics and the effective ultrafiltration achieved could prevent both excessive and insufficient fluid removal. Excessive removal may compromise the patient’s hemodynamics and lead to unnecessary increases in the drug dosage, tissue hypoperfusion (especially in vasculopathic and elderly patients), and hyperlactatemia. Similarly, delayed initiation of the resuscitation phase may prolong recovery time, the ventilatory support time, compartment syndrome, as well as excessive and unnecessary RRT time.

Other complications related to RRT include the removal of nutrients (amino acids, minerals, vitamins, carbohydrates, essential elements – such as selenium), and drugs. The patient’s diet should be adjusted accordingly. In some cases, euglycemic ketoacidosis may occur^74^.

The pharmacokinetics of various drugs, especially antibiotics, are altered by RRT methods. Continuous modalities more efficiently remove several antibiotics, which should be administered in usual doses, not reduced according to renal function (meropenem, vancomycin). Whenever possible, serum levels should be monitored. Intermittent modalities, on the other hand, require the administration of additional doses following the procedure. These therapeutic aspects should be familiar to the nephrologist, who should guide the assisting team on the matter. As Therapeutic Drug Monitoring is not available for most antibiotics, dose adjustments should be made based on the therapy dose, method, and frequency of RRT. Some recently published correction tables are available for consultation^75^.

### Quality and Safety

It is recommended that Nephrology services providing dialysis support routinely assess the quality of the services provided. Several parameters may be used, such as the time between therapy indication and initiation, the consumption of supplies (filters, solutions, and catheters), the effective dose of therapy and ultrafiltration, equipment usage time, the number of treatment-related complications, and problem-solving capacity^76,77^.

Primary outcomes including mortality, length of hospital stay, length of ICU stay, recovery of kidney function, and referral for chronic dialysis therapy should also be monitored^76,77^.

It is recommended that the recent ANVISA regulations, standardized for hemodialysis services and governing patient safety practices, be followed by services that provide inpatient RRT. In this regard, the following indicators should be assessed on a monthly basis: patient safety plan; hand hygiene; patient identification; fall prevention; safety protocol for prescribing and administering medication; prevention of adverse events related to vascular access; prevention of infections and adverse events in peritoneal dialysis; prevention of system clotting during extracorporeal therapy; prevention and control of transmission of resistant microorganisms, as well as HIV and hepatitis B and C; implemented protocol for monitoring the quality of hemodialysis water; technology (equipment) management; and reporting of incidents related to staff members^78,79^.

Water quality assessment and infection control related to nephrological care are mandatory for hospital-based Nephrology services and will be described below^54,58,78,79^.

### Water Control

The parameters for the collection and microbiological analysis of potable and treated water, as well as the maintenance procedures for the storage system, must comply with the provisions established in the ANVISA Resolution RDC No. 11, dated March 13, 2014^58,78^.

The schedule for the collection and microbiological analysis of potable and treated water must be established by the Hospital Infection Control Service (HICS).

The hospital must maintain daily and monthly records of the analysis of physical, organoleptic, and microbiological characteristics of the potable water supplied for dialysis therapy.

The Nephrology service must maintain monthly and semi-annual records of the analysis of treated water, with its microbiological and physicochemical characteristics, as well as the schedule for disinfecting and replacing equipment membranes and filters.

### Infection Control

In order to reduce healthcare-related infections in dialysis patients, hospitals should, through the HICS, provide professional training and specific records focusing on: cleaning and disinfection of equipment; care in handling hemodialysis and peritoneal dialysis catheters, as well as insertion and maintenance routines; measures adopted during episodes of bacteremia in dialysis sessions; guidance on cleaning reusable items and surfaces, as well as on waste and material disposal; actions regarding water quality reports that do not meet the required standards; guidance on the use of PPE by professionals; instructions on the procedures that should be adopted for patients colonized by MD bacteria or those with transmissible respiratory diseases^58,77,78,79^.

### Records

Records of care provided by the multidisciplinary team in Nephrology should be entered into the hospital’s standard medical records. The medical record should include: clinical history, daily assessment, justification for the initiation of dialysis therapy, choice of modality, methods, and type of access. In addition, medical assessments conducted prior to the initiation of procedures should be included, as well as guidance regarding any parameter changes during therapies.

Medical prescriptions must be provided in detail prior to the initiation of each therapy. Any necessary adjustments must be recorded in a medical record or standardized document, duly signed and stamped by the responsible healthcare professional.

The nursing and technician records, in the medical charts, should include routine assessments, an operational description of the initiation, maintenance, and termination of therapies, as well as the reporting of events and complications.

Aspects relating to access, such as handling, and maintenance must also be properly recorded.

## Outpatient follow-up of Patients with Acute Kidney Injury/Disease

Patients with AKI, especially those admitted to the ICU, exhibit high morbidity and mortality in the months following hospital discharge. There is a high risk of clinical complications and hospital readmissions, particularly in patients with diabetes, cardiovascular disease, and CKD^80, 81, 82^.

Patients with AKI may present the following progression outcomes:

a. Immediate resolution within 7 days;
b. Immediate resolution, with relapses occurring within 90 days;
c. Late resolution within 90 days;
d. No resolution within 90 days, progressing to CKD.

Acute Kidney Disease (AKD) is the persistence of acute or subacute renal dysfunction/injury, as manifested by KDIGO AKI stage ≥ 1, between 7 and 90 days following an episode of AKI^83,84^.

Patients with AKI, even at early stages, are at greater risk for cardiovascular complications, death, and the development of CKD^84^.

It is therefore recommended that patients surviving an AKI episode receive the following care after hospital discharge:

1. Outpatient follow-up by the attending physician, with evaluation of renal function: serum creatinine and albuminuria measurements, no later than 3 months after discharge;
2. When elevated creatinine and/or albuminuria persist 3 months after an aki event, these patients should be followed up by nephrologists, in accordance with ckd guidelines;
3. In patients with complete recovery from aki, periodic renal evaluation (serum creatinine and albuminuria measurements) is recommended, at least one year after hospital discharge;
4. Patients with persistent aki, other nephropathies, and ckd should be referred to a nephrologist for outpatient follow-up.

## Data Availability

All data and bibliography utilized for this manuscript are available with authors.
