# Supplementary material for: Guidelines for hospital nephrology assistance from the Brazilian Society of Nephrology (BSN)
Source: J Bras Nefrol. 2025 May 30;47(3):e20240239. doi: 10.1590/2175-8239-JBN-2024-0239en (PMC12124864; doi:10.1590/2175-8239-JBN-2024-0239en)
Supplement: Translation

# Translation: Guia de assistência nefrológica hospitalar da Sociedade Brasileira de Nefrologia (SBN)

**DOI:** 10.1590/2175-8239-JBN-2024-0239pt

**Authors:** Mauricio Younes-Ibrahim, Eduardo Rocha, Thiago Reis, Vinícius Sardão Colares, Emerson Quintino de Lima, Lucia da Conceição Andrade, Eduardo Cantoni Rosa, Helen Siqueira Cardoso, Fernando Thomé, Daniela Ponce, José H. Rocco Suassuna, Luis Yu

**Affiliations:** 1 Sociedade Brasileira de Nefrologia, Departamento de Injúria Renal Aguda, São Paulo, SP, Brasil.; 2 Universidade Estadual de São Paulo, Departamento de Clínica Medica, Serviço de Nefrologia, São Paulo, SP, Brasil.

**Keywords:** Sociedade Brasileira de Nefrologia, Guia de Prática Clínica, Assistência Hospitalar, Nefrologia, Injúria Renal Aguda, Diálise Renal

## Abstract

O Departamento de IRA da Sociedade Brasileira de Nefrologia (SBN) elaborou um guia de assistência nefrológica hospitalar, que abrange os aspectos envolvidos na atuação do nefrologista no atendimento a pacientes portadores de doenças renais dentro do ambiente hospitalar. O guia aborda os seguintes tópicos principais: 1) o papel do nefrologista na assistência hospitalar; 2) a terapia de suporte renal não-dialítico; 3) as normas técnicas para o atendimento dialítico hospitalar; e 4) o seguimento ambulatorial de pacientes com injúria/doença renal aguda. O guia descreve detalhadamente as principais atribuições do nefrologista, sua atuação no atendimento hospitalar não-dialítico e dialítico, além de descrever todos os métodos dialíticos disponíveis, a infraestrutura necessária, os recursos humanos e os registros destes procedimentos. O guia finaliza com normas para o seguimento ambulatorial dos pacientes nefrológicos após a alta hospitalar. A finalidade precípua deste guia da SBN é fornecer subsídios para um melhor atendimento médico e multiprofissional aos nefrologistas e outros profissionais envolvidos na assistência hospitalar de pacientes nefrológicos.

## Papel do Nefrologista na Assistência Hospitalar

A Nefrologia é uma especialidade médica formalmente estabelecida na década de 1960 e reconhecida pela Associação Médica Brasileira (AMB). Para que um médico seja considerado especialista em nefrologia, é necessário obter o título de especialista, por meio do Ministério da Educação e/ou Sociedade Brasileira de Nefrologia/AMB, devidamente registrado no respectivo Conselho Regional de Medicina.

A atuação do nefrologista no ambiente hospitalar é ampla e as competências da especialidade englobam:

1. Consultoria nos projetos de instalação e utilização de equipamentos específicos da especialidade
2. Definição dos protocolos da linha de cuidados para nefrologia
3. Coordenação das atividades da equipe multiprofissional envolvida no atendimento nefrológico
4. Disponibilidade para emitir pareceres nefrológicos em auxílio aos demais médicos do corpo clínico do hospital
5. Atualização permanente dos novos conhecimentos e novas tecnologias em nefrologia, para serem empregados no contexto hospitalar
6. Participação do nefrologista em comissões de qualidade e segurança que visem estabelecer e disseminar iniciativas de prevenção e reconhecimento precoce de piora da função renal
7. Papel do nefrologista como educador e disseminador das melhores práticas para o manejo destes pacientes, considerando que muitos pacientes internados com injúria renal aguda (ira) serão cuidados por médicos não-especialistas
8. Responsabilidade pelo atendimento e pelos cuidados nefrológicos aos pacientes hospitalizados, contemplando:
  Identificação de pacientes com risco de desenvolver comprometimento da função renal;
  Medidas de prevenção e proteção para afecções renais;
  Adequação individualizada das prescrições de medicamentos para as respectivas funções renais, com vistas à prevenção de eventos de nefrotoxicidade;
  Prevenção e monitoramento de complicações de procedimentos intra-hospitalares com potenciais impactos sobre a função renal;
  Definição e emprego dos métodos complementares pertinentes para auxiliar e detalhar os diagnósticos das doenças renais;
  Indicação dos métodos de terapia de suporte renal artificial (sra);
  Prescrição e supervisão dos procedimentos de sra;
  Providências relativas aos acessos vasculares e peritoneais necessários para os sra;
  Condução das terapias contínuas de sra em pacientes críticos, em conjunto com as equipes médicas de unidades fechadas;
  Interação com equipes multidisciplinares para a definição de estratégias terapêuticas comuns;
  Atividades clínicas de preparação e realização de transplantes renais;
  Preparo e encaminhamento para continuidade de acompanhamento nefrológico e/ou métodos de depuração após desospitalização;
  Identificação criteriosa dos casos selecionados para a adoção de cuidados paliativos.
9. A presença do nefrologista no ambiente hospitalar é essencial para a prescrição e acompanhamento dos procedimentos nefrológicos. o uso de telemedicina para atividade nefrológica hospitalar remota aplica-se apenas em casos de teleconsultoria interdisciplinar à equipe médica que assiste ao paciente, não sendo aplicável para o exercício de teleconsultas aos pacientes internados ou à prescrição de terapia renal substitutiva
10. Relação trabalhista (serviços de nefrologia públicos e privados)
  A sociedade brasileira de nefrologia recomenda que cada hospital tenha, devidamente identificada, uma equipe permanente de médicos nefrologistas, própria ou contratada, que funcione continuamente integrada ao seu corpo clínico e que exerça plenamente as atribuições da especialidade. as múltiplas competências discriminadas acima requerem pactuações específicas das respectivas remunerações.

## Terapia De Suporte Renal Não-Dialítico

O nefrologista, integrante obrigatório do corpo clínico hospitalar, é o profissional capacitado para fornecer cuidados a pacientes com disfunção renal, distúrbios hidroeletrolíticos e ácido-base. Este especialista também possui competências para elaborar estratégias de prevenção de IRA, interagir com a equipe multidisciplinar e para redigir protocolos para padronização do cuidado que abrange a maioria dos casos, ao mesmo tempo em que mantém flexibilidade para individualizar o cuidado em situações peculiares. Neste sentido, é fundamental a participação do nefrologista em comissões de qualidade e segurança que tenham como objetivo elaborar estratégias de prevenção de nefrotoxicidade, realizar a avaliação clínica pré-operatória de pacientes com alteração da função renal ou abordar distúrbios eletrolíticos e ácido-base^1^.

Pacientes internados com IRA serão também assistidos por médicos não nefrologistas. Por esse motivo, é importante o papel do nefrologista como educador e disseminador das melhores práticas para o manejo destes pacientes. As estratégias de suporte não-dialítico da IRA são semelhantes às utilizadas na sua prevenção^1^.

### Os Nefrologistas Devem Atuar em Diversas Frentes, Visando Proporcionar Tratamento não-dialítico no Ambiente Hospitalar, Incluindo

1. Atuação junto aos gestores hospitalares e como coordenador do serviço de nefrologia local;
2. Dedicação ao apoio à infraestrutura^1^;
3. Envolvimento com as lideranças de várias unidades, a fim de alinhar o cuidado, desenvolver e supervisionar avaliações de qualidade, além de criar uma cultura de sucesso atrelada às ações da nefrologia^1-3^;
4. O apoio da liderança hospitalar é essencial para essa atuação^1,2^.

### Participação em Comissões de Qualidade e Segurança

Embora parte dos casos de IRA seja potencialmente evitável, essa condição apresenta um elevado impacto nos custos hospitalares e na sobrevida de pacientes hospitalizados^4-6^. Dessa forma, o desenvolvimento de IRA pode ser considerado um indicador de qualidade do atendimento hospitalar. A inclusão do nefrologista em comissões de qualidade e segurança pode auxiliar a estabelecer e disseminar iniciativas de prevenção e reconhecimento precoce de disfunção renal^1,7,8^.

### Avaliação pré-Operatória

Os pacientes portadores de doença renal crônica (DRC) apresentam risco elevado de complicações no perioperatório^9^. É recomendada a consulta nefrológica na avaliação pré-operatória destes pacientes e daqueles com alto risco de disfunção renal no pós-operatório. O acompanhamento desses pacientes é crucial para intervenções precoces em fatores de riscos modificáveis e monitoramento dos não-modificáveis^10^. Para os pacientes que já se encontram em suporte dialítico, o atendimento nefrológico é obrigatório, com vistas à organização do melhor momento para prescrição da diálise nos períodos pré e pós-operatório. Pacientes que não apresentam DRC, mas têm alto risco de desenvolver alterações renais durante o procedimento cirúrgico, como pacientes idosos com múltiplas comorbidades, aqueles em uso de diversos medicamentos, pacientes criticamente enfermos e os com insuficiência cardíaca congestiva grave e hepatopatias, deveriam também ser acompanhados pelo serviço de Nefrologia^10^.

### Cuidados de Pacientes com Doença Renal Crônica

Os pacientes hospitalizados com DRC, incluindo não-dialíticos, dialíticos e transplantados renais, devem ser acompanhados pelo nefrologista, a fim de identificar e corrigir interações medicamentosas, ajustar os medicamentos conforme o grau de disfunção renal, evitar drogas nefrotóxicas e estabelecer medidas de prevenção de nefrotoxicidade e o manejo de drogas imunossupressoras^10^. Estas atividades podem ser desenvolvidas em conjunto com o farmacêutico clínico, o nutricionista e o médico assistente, para estabelecer a dieta mais adequada, o manejo volêmico, a correção de distúrbios eletrolíticos e ácido-base, gerenciar o acesso vascular e otimizar o suporte dialítico.

### Cuidados de Pacientes com IRA

Muitos pacientes internados com IRA serão cuidados por médicos não especialistas, reforçando a relevância do nefrologista como educador e disseminador das melhores práticas para o manejo destes pacientes^1^. Além das atividades já descritas, suas atribuições incluem: o reconhecimento precoce da disfunção renal, a elaboração de estratégias de prevenção da nefrotoxicidade, o controle da volemia, a identificação e correção dos distúrbios hidroeletrolíticos e ácido-base, bem como o ajuste de drogas para o grau de disfunção renal.

A atuação do nefrologista no tratamento não dialítico de pacientes com IRA envolve também o controle adequado das seguintes manifestações clínicas:

#### Hemodinâmica e Fluidos

1. Em caso de hipovolemia, administrar soluções de hidratação adequadas;
2. A meta de reanimação inicial, particularmente em pacientes com sepse, é manter a pressão arterial média (pam) acima de 65 mmhg. em pacientes previamente hipertensos, alvos mais elevados podem ser considerados^11-16^;
3. O uso de vasopressores pode ser necessário para atingir as metas de pam. a noradrenalina é o vasopressor mais utilizado para pacientes com ira por sepse. em pacientes em uso de noradrenalina com dificuldade de atingir a pam alvo, a vasopressina pode ser associada^13,17^;
4. Monitorar a pressão intra-abdominal (pia) em casos selecionados. a perfusão renal depende da diferença entre a pressão de entrada (pam) e saída (pressão venosa central – pvc). pvc elevada e congestão venosa renal estão associadas à ira em pacientes com insuficiência cardíaca e síndrome compartimental abdominal. neste último caso, a pia pode estar maior que a pvc, e a pressão de perfusão renal passa a ser a diferença entre a pam e a pia^13,17^;
5. Evitar expansão volêmica desnecessária, controlando a administração de soros de manutenção e diluições excessivas de medicações, além de desestimular a ingestão hídrica excessiva, especialmente em pacientes oligúricos^18-22^;
6. Faz-se necessário o controle rigoroso da diurese e principalmente do fluxo de diurese^23^;

#### Uso de Diuréticos

1. Administrar furosemida no tratamento de hipervolemia e hipercalemia. outros diuréticos (tiazídicos, acetazolamida, espironolactona, amilorida) podem ser associados para duplo ou mesmo triplo bloqueio dos segmentos do néfron^24^;
2. Não retardar a tomada de decisão sobre diálise, especialmente quando não houver resposta à administração de diuréticos; mesmo que haja alguma resposta aos diuréticos, a diálise deve ser considerada se houver piora da função renal^25^;
3. O “teste de stress com furosemida” (infusão de 1 mg/kg de furosemida em bolus em pacientes considerados euvolêmicos ou hipervolêmicos, sem uso prévio de diurético, ou 1,5 mg/kg em pacientes com uso prévio de diurético) é uma forma de identificar os pacientes com maior risco de progressão da ira e necessidade de diálise. são aqueles que não respondem com diurese > 200 ml em 2 horas após a infusão do diurético^26^;

#### Nutrição

1. Deve-se procurar atingir a ingestão de 20-30 kcal/kg/dia em todos os estágios de ira^27^;
2. Evitar a restrição da ingestão de proteínas com o objetivo de prevenir ou retardar o início da diálise^27,28^;
3. Administrar 0,8-1,0 g/kg/dia de proteínas em pacientes com ira sem necessidade de diálise e 1,0-1,5 g/kg/dia em pacientes em diálise, até um máximo de 1,7 g/kg/dia em pacientes em diálise contínua ou hipercatabólicos^28^;
4. Evitar episódios de hiperglicemia, procurando manter a glicemia abaixo de 180 mg/dl. o controle glicêmico é indispensável nos cuidados de pacientes críticos e cirúrgicos, pois tanto a hiperglicemia quanto a hipoglicemia estão relacionadas ao aumento da mortalidade e à ocorrência de ira. o kdigo recomenda manter a glicemia entre 110 e 149 mg/dl, com estreita vigilância para evitar a hipoglicemia^29,30^;
5. Atenção à ingestão ou administração de dietas com conteúdos excessivos de sódio, potássio e fósforo, bem como à restrição hídrica, quando necessária;

#### Acidose e Hipercalemia

1. Caso o ph sanguíneo seja < 7,2, particularmente quando associado a bicarbonato sérico inferior a 15 meq/l, e sem evidência de hipervolemia, pode-se administrar bicarbonato de sódio^31^;
2. Atenção a outras causas de acidose metabólica que não sejam devido à disfunção renal, tais como: intoxicações exógenas, acidose láctica e cetoacidose diabética^31^;
3. Ao corrigir a acidose metabólica, pode ocorrer queda do cálcio ionizado. se necessária a correção do cálcio, deve-se infundir em linha venosa separada, devido à incompatibilidade das soluções de cálcio e bicarbonato^31^;
4. O tratamento da hipercalemia, principal emergência nefrológica, deve ser prontamente instituído, incluindo-se medidas de proteção da membrana miocárdica (gluconato de cálcio), medidas de redistribuição do potássio extra para o intracelular (solução de glicose/insulina, b2 adrenérgicos e bicarbonato de sódio) e remoção do potássio corporal (furosemida, resinas e ligantes de troca iônica e diálise). a hipercalemia é uma condição extremamente grave que pode levar ao óbito; portanto a diálise deve ser instituída rapidamente quando não houver resposta às medidas clínicas^32^;

#### Anemia

1. Hemoglobina < 8 mg/dl é um fator de risco para ira, tanto no pré quanto no pós-operatório, para a maioria dos procedimentos cirúrgicos. recomenda-se a otimização dos níveis de hemoglobina no pré-operatório para evitar transfusões sanguíneas e descompensações no pós-operatório^33^.

## Normas Técnicas do Atendimento Dialítico Hospitalar

O disposto neste guia aplica-se aos serviços de Nefrologia que oferecem SRA, diálise peritoneal e outros métodos de depuração extracorpórea, procedimentos considerados de alta complexidade, realizados em ambiente hospitalar^34^.

### Indicações

As indicações do SRA serão aquelas consideradas de urgência ou clássicas (acidose, distúrbios eletrolíticos, intoxicações medicamentosas, sobrecarga hídrica e uremia) ou ainda aquelas baseadas na demanda (nutrição, drogas, hemoderivados) *versus* reserva renal, avaliadas através da evolução da trajetória de escórias, sobrecarga volêmica, acometimento de outros órgãos e da necessidade da remoção inflamatória no contexto do paciente crítico^35-37^.

O nefrologista deve, ainda, considerar a natureza do insulto e a probabilidade de recuperação, a doença básica, as comorbidades, as considerações éticas e os riscos do procedimento^38-44^.

### Modalidades/Métodos Extracorpóreos

#### Descrição

Considerando os mecanismos de troca de solutos (difusão, convecção e ultrafiltração), preferencialmente utilizados nas três modalidades dialíticas de escolha (convencional, prolongada e contínua), serão designados os diferentes métodos a serem aplicados no paciente renal hospitalizado (hemodiálise, hemofiltração, hemodiafiltração e ultrafiltração) (Tabela 1)

**Tabela 1:** Métodos: modalidades, mecanismos e características

		Modalidades		
		SRA Convencional (HDC)	SRA Prolongado (HDP)	SRA Contínuo (TRSC)
Mecanismos de troca de solutos	Difusão	Hemodiálise	Hemodiálise	Hemodiálise
	Convecção	–	–	Hemofiltração
	Difusão/Convecção	–	Hemodiafiltração	Hemodiafiltração
	Ultra filtração	Ultrafiltração	Ultrafiltração	Ultrafiltração
Cinética - Eficiência		Alta	Média	Baixa
Depuração de drogas/toxinas por unidade de tempo		Maior	Intermediário	Menor
Cinética - Eficácia semanal, remoção de escórias/inflamação ao longo do tratamento		Menor	Intermediária	Maior
Instabilidade hemodinâmica		Maior	Intermediária	Menor
Aumento PIC		Maior	Intermediário	Menor
Controle volemia		Menor	Intermediário	Maior
Anticoagulação		Dispensável	Dispensável	Sim/eventualmente dispensável
Compatibilidade com outros procedimentos extracorpóreos		Não	Não	Sim
Transporte do paciente*		Sim	Sim	Não
Custo		Menor	Intermediário	Maior

Abreviaturas – PIC: pressão intracraniana; HDC: hemodiálise convencional; HDP: hemodiálise prolongada; TRSC: terapia renal substitutiva contínua. Nota – *A mobilização do paciente (sentar-se em poltrona, por exemplo) depende da localização do acesso vascular e da estabilidade hemodinâmica, mas a saída do leito, para intervenções e exames, é facilitada nas modalidades intermitentes.

Na modalidade de hemodiálise convencional (HDC), a utilização de troca de solutos na diálise hospitalar dá-se preferencialmente por método difu­sivo, em regime de alta eficiência, predispondo assim à maior flutuação osmótica e de balanço hídrico e, consequentemente, à maior instabilidade hemo­dinâmica e potencial aumento na pressão intracraniana (expansão do compartimento intracelular)^45,46^.

Na modalidade de hemodiálise prolongada ou estendida (HDP), as flutuações osmóticas e de balanço hídrico serão intermediárias, ainda com predisposição à instabilidade^47^.

Na terapia renal substitutiva contínua (TRSC), a remoção de líquidos ocorre de forma mais lenta, a cinética de troca de solutos é baixa e há a possibilidade de combinação com método convectivo, permitindo uma maior estabilidade e adequação aos pacientes mais graves, em especial àqueles com tendência à hipertensão intracraniana, disnatremias e hipercatabolismo^48,49^.

A ultrafiltração destina-se ao equilíbrio volêmico do paciente e poderá ser indicada isoladamente ou em associação a outros métodos nas diferentes modalidades (Tabelas 1 e 2)^50^. As características das modalidades extracorpóreas de SRA na IRA estão resumidas na Tabela 1 ^51-53^.

**Tabela 2:** Parâmetros operacionais nas modalidades dialíticas

	HD Convencional	HD Prolongada	TRSC Contínua
Setor	Sala hemodiálise, Unidade de Internação UTI, Semi UTI	UTI, Semi UTI	UTI
Método	Hemodiálise Ultrafiltração isolada	Hemodiálise Hemodiafiltração Ultrafiltração isolada	Hemodiálise Hemofiltração Hemodiafiltração
Acesso	FAV, cateter temporário ou permanente	FAV, cateter temporário ou permanente	Cateter temporário ou permanente
Equipamento	Máquina de hemodiálise	Máquinas de hemodiálise ou de terapia contínua	Máquina de terapia contínua
Dispositivo	Membrana com polímero biocompatível	Membrana com polímero biocompatível	Membrana com polímero biocompatível
Tempo de sessão	até 6 h	de 6 a 12 h	24 h
Periodicidade	3 a 4x/semana ou diária	3 a 4x/semana ou diária	Contínua
Fluxo Dialisante	300–800 ml/min	100–300 ml/min	Variável, usualmente 20–35 ml/Kg/h (incluindo solução de reposição quando houver convecção)
Fluxo Sangue ml/min	200–400	100–250	100–200 ml
Ultrafiltração efetiva	0–4 l	0–4 l	Variável, conforme demanda
Anticoagulação	Com ou sem heparina (não fracionada ou hbpm)	Com ou sem heparina (não fracionada ou hbpm); citrato regional	Com ou sem heparina (não fracionada ou hbpm); citrato regional
KT/V alvo (sessão/sem)	0,9 a 1,3/2,0 a 2,5	0,9–1,0/3,0–3,5	20–30 ml/kg/h*

Abreviaturas – FAV: fístula arteriovenosa; hbpm: heparina de baixo peso molecular. Nota – *A dose de TRS contínua engloba o fluxo total de efluente (solução de dialisato, hemofiltração + ultrafiltração, incluindo, em alguns equipamentos, a filtração compensatória à infusão de citrato ou cálcio).

### Escolha Da Modalidade

A escolha da modalidade dialítica é individualizada, levando em consideração o quadro clínico de cada paciente, bem como a disponibilidade de equipamentos e a capacitação da equipe nos diferentes métodos. Essa decisão deve ser tomada pelo nefrologista, em acordo com o intensivista ou médico assistente do paciente (Tabela 1)^54^.

### Diálise Peritoneal

A diálise peritoneal (DP) é uma opção terapêutica para o tratamento de pacientes portadores de IRA, que ganhou destaque novamente após a pandemia de COVID-19. Demonstra, em alguns estudos, não inferioridade quanto aos desfechos em pacientes com IRA, quando comparada à terapia extracorpórea^55^.

Em casos selecionados, a DP pode ser uma opção mais adequada, como, por exemplo, quando há contraindicação à anticoagulação, nos casos em que há dificuldades no acesso vascular, em estados de hipercoagulabilidade, após cirurgia cardíaca, em situações de limitação de recursos locais e em pediatria. Além disso, a DP não provoca síndrome de desequilíbrio, corrige gradualmente os desequilíbrios, é mais biocompatível e é adequada para situações de síndrome cardiorrenal e pacientes neurocríticos^56,57^. Entretanto, é contraindicada em comunicação pleuroperitoneal, insuficiência respiratória e diafragmática severa, hipercalemia com risco de vida não responsiva, hipercatabolismo severo, baixa depuração peritoneal, celulite da parede abdominal, grandes hérnias/aderências/fibrose peritoneal, íleo adinâmico, cirurgia abdominal recente ou outras alterações abdominais confirmadas ou suspeitas. Além disso, não é segura para o tratamento de edema agudo de pulmão.

Os tipos de diálise peritoneal são variados, permitindo flexibilidade terapêutica: intermitente (com tempos de permanência curtos, duração até 24 horas, repetida conforme a necessidade); equilibrada contínua (com tempos de permanência mais prolongados, em ciclos contínuos); corrente ou “tidal” (semelhante à intermitente, mas com volume peritoneal residual mantendo equilíbrio durante os ciclos); e de alto volume (com volumes diários totais elevados). O volume de cada ciclo depende do tamanho do paciente, podendo variar de 1 a 3 litros no adulto e 10-50 ml/Kg em crianças. Há grande variação de práticas, dependendo dos serviços individuais.

A dose mais apropriada para a DP no manejo de pacientes com IRA ainda está mal definida. Essa falta de uma definição clara ocorre devido ao número limitado de estudos disponíveis para comparar o tratamento pelas diferentes modalidades. Embora o Kt/V seja considerado ideal, ele não é prático. A adequação é, na prática, analisada por sinais clínicos e balanço hídrico, normalização dos níveis de potássio e melhoria do padrão ácido-base.

### Normas Técnicas

1. No intuito de prover a melhor assistência dialítica aos pacientes internados, recomendase que os corresponsáveis, incluindo hospitais e serviços de Nefrologia, cumpram as orientações normativas dispostas neste guia;
2. Recomenda-se que os hospitais forneçam suporte de Nefrologia e SRA à beira leito para os pacientes que necessitem de SRA e não tenham condições de remoção para unidades de diálise ambulatorial, seja intra ou extra hospitalar. Além disso, é necessário que os hospitais providenciem as melhores condições de infraestrutura física nas unidades hospitalares (UTI ou unidades de internação) para realização de SRA^58^;
3. Compete ao serviço de Nefrologia a indicação, instalação, monitoramento e término do tratamento dialítico aos pacientes internados, em conformidade com os protocolos e procedimentos operacionais padrão pré-estabelecidos junto ao hospital^58^;
4. Cabe aos corresponsáveis, conforme o contrato estabelecido, fornecer os equipamentos, materiais e medicações necessários ao tratamento de SRA.

### Equipe Multiprofissional

O serviço de Nefrologia que presta assistência dialítica deverá ser composto por profissionais legalmente habilitados, que atendam às competências específicas para os processos aos quais foram designados. Deve ser supervisionado por um médico nefrologista responsável técnico (RT) e um RT de enfermagem, ambos titulados em Nefrologia pelos respectivos Conselhos de Classe ou Sociedade da Especialidade, e que assumam a responsabilidade técnica perante a autoridade sanitária pelo serviço de saúde, conforme legislação vigente.

Esses profissionais também deverão elaborar, implementar e monitorar os procedimentos operacionais e protocolos estabelecidos, juntamente às demais equipes médicas e não médicas do Serviço de Nefrologia e do hospital. Além disso, serão responsáveis pela escala de profissionais e deverão participar das reuniões junto ao hospital, relacionadas à segurança dos pacientes, controle de infecções e tratamento de água.

Também deverão compor o processo assistencial outros profissionais, incluindo médicos nefrologistas, intensivistas e hospitalistas, enfermeiros especialistas em Nefrologia, técnicos de enfermagem com capacitação nos diferentes métodos de TRS, cirurgião vascular e geral, farmacêutico e técnico de equipamentos, cada um com atribuições específicas, a saber:

– Médicos nefrologistas: avaliação presencial dos pacientes; indicação do tratamento sob demanda do hospitalista ou intensivista; prescrição da terapia de SRA; prevenção de complicações do procedimento; instalação ou solicitação do acesso vascular; alteração dos parâmetros dialíticos quando necessário (fluxos, concentrações de eletrólitos nas soluções, ajuste da anticoagulação, troca de modalidade ou métodos). Recomenda-se que o nefrologista RT determine, em cada contexto, a necessidade da presença ou supervisão nefrológica durante a condução de terapias intermitentes, bem como a carga de trabalho que o nefrologista poderá atender^54,59^.
– Pediatra: recomenda-se que o atendimento pediátrico para SRA seja realizado por um nefrologista pediátrico. Em serviços onde não houver nefrologista pediátrico disponível, o SRA ou a diálise peritoneal à beira leito poderão, eventualmente, ser acompanhados por um nefrologista de adultos e um pediatra, ambos aptos a atender intercorrências clínicas.
– Médicos hospitalistas ou intensivistas: atendimento de intercorrências clínicas; eventual instalação de acesso vascular e avaliação do seu correto posicionamento; ajuste da intensidade de ultrafiltração, conforme necessário e em concordância com o nefrologista.
– Médico Cirurgião Vascular e Geral: instalação de acesso vascular ou peritoneal.
– Enfermeiro nefrologista: instalação e finalização das terapias contínuas; suspensão temporária do tratamento por intercorrência; cuidados com o acesso vascular, inclusive tracionamento ou troca de vias do acesso vascular quando necessário, e punção de fístula arteriovenosa quando indicado.
– Enfermeiro da UTI: acompanhamento dos procedimentos e suas intercorrências, especi­almente das terapias contínuas e da finalização do tratamento. Quando capacitados em Nefrologia, podem substituir ou trabalhar em cooperação com a enfermagem da Nefrologia. É vetada a punção de fístulas ou enxertos arteriovenosos por enfermeiro não capacitado para tal.
– Técnico de Enfermagem da Nefrologia: instalação de SRA convencional e prolongado; acompanhamento dos procedimentos à beira leito; instalação de SRA contínuo sob supervisão de enfermeiro; punção de fístula arteriovenosa, quando tiver treinamento e prática para tal, sob a determinação e orientação do enfermeiro.
– Farmacêutico: acompanhamento das doses de fármacos prescritas, ajustes conforme a função renal e o tratamento em uso, supervisão do preparo de soluções para TRSC em centros onde exista a possibilidade de preparo destas.
– Técnico de Equipamentos: manutenção preventiva ou corretiva dos equipamentos de SRA e de purificação da água.

### Infraestrutura–Unidades Hospitalares

O hospital deve disponibilizar, junto aos leitos, nas unidades de internação, de terapia intensiva e semi-intensiva: ponto de água potável adaptado; ponto de esgoto para o expurgo de efluentes; rede elétrica com adaptações para instalação dos equipamentos de diálise e osmose.

As máquinas de diálise, assim como as de osmose reversa, além dos insumos utilizados, devem ter seus registros regularizados junto ao Ministério da Saúde e aprovados junto à Anvisa. Devem ser operadas de acordo com as recomendações do fabricante.

Além disso, materiais e equipamentos para atendimento de emergência devem estar disponíveis de forma acessível e em perfeitas condições de funcionamento, próximos aos locais de atendimento dialítico.

O hospital deve, ainda, disponibilizar local específico para a guarda, limpeza e desinfecção de equipamentos e materiais, bem como para o descarte de agulhas, linhas, filtros, isoladores de pressão e sobras de soluções, que não deverão ser reutilizados. Tal local deve permanecer limpo, ventilado e protegido da luz, do calor e da umidade, devendo conter ponto de água potável, ponto de drenagem, tomada para equipamentos e pia para higienização das mãos^54,58^.

### Parâmetros Operacionais Nas Terapias

O Serviço de Nefrologia deve, preferencialmente, dispor de diferentes modalidades e métodos dialíticos, que possam ser adequados às diversas situações clínicas apresentadas pelos pacientes acometidos de IRA e por aqueles com DRC estágio V. Recomenda-se que as modalidades extracorpóreas e seus respectivos métodos (técnicas), assim como a DP, sejam disponibilizadas pelo Serviço de Nefrologia dos hospitais.

A Tabela 2 apresenta os parâmetros operacionais habitualmente utilizados nas modalidades e métodos mais empregados na condução de pacientes com quadro de IRA^60,61^.

A utilização de métodos menos rotineiros, como a plasmaférese terapêutica, a hemoperfusão e o suporte hepático artificial, também poderão ser de responsabilidade da equipe de Nefrologia e está ilustrada na Tabela 3 ^62-65^.

**Tabela 3:** Métodos extracorpóreos

	Hemoadsorção	Plasmaférese terapêutica por membrana	Sistema de recirculação para adsorção molecular (MARS)	Diálise com albumina de passagem única (SPAD)	Remoção extracorpórea de dióxido de carbono (ECCO2R)
Alvo-remoção	Moléculas médias, fármacos, toxinas, endotoxinas, bilirrubinas e sais biliares	Anticorpos, toxinas, imunocomplexos, elementos figurados do sangue	Bilirrubina e sais biliares	Bilirrubina e sais biliares	Dióxido de carbono
Setor	Sala de hemodiálise, unidade Internação, UTI, Semi UTI	Unidade Internação, UTI, Semi UTI	UTI, Semi UTI	Sala de hemodiálise, unidade Internação, UTI, Semi UTI	UTI
Método	UTI, Semi UTI	Unidade Internação, UTI, Semi UTI	UTI, Semi UTI	Sala de hemodiálise, unidade Internação,	Hemodiálise
Acesso	UTI, Semi UTI	UTI	Cateter temporário, permanente	Cateter temporário, permanente	Cateter temporário, permanente
Equipamento (máquinas)	Hemodiálise, terapia contínua, específicas para hemoperfusão; cartuchos acoplados circuitos extracorpóreos	Hemodiálise, terapia contínua com módulo para plasmaférese	Específica acoplada à máquina de hemodiafiltração contínua ou hemodiálise	Terapia contínua	Específica para hemoperfusão, terapia contínua acoplada à membrana oxigenadora
Dispositivos	Cartuchos com resinas ou microesferas	Filtros de membrana com polímero biocompatível	Filtros de membrana com polímero biocompatível, circuito de carvão ativado e cartucho troca iônica	Filtros de membrana com polímero biocompatível	Membrana de remoção de CO2
Tempo de sessão	2–24 h	2 h	6–8 h	7–8 h com albumina + 15–16 h hemodiafiltração	72 h
Periodicidade	Diária	Diária; a cada 48hs	Diária	Diária	Diária
Tempo de tratamento	72 h ou mais	Variável	72 h ou mais	72 h ou mais	72 h ou mais
Anticoagulação	Heparina, citrato ou sem anticoagulação	Heparina, citrato ou sem anticoagulação	Heparina, citrato ou sem anticoagulação	Heparina, citrato ou sem anticoagulação	Heparina, ou sem anticoagulação
Solução específica	Não	Albumina com solução fisiológica ou plasma	Solução com albumina	Solução para hemodiafiltração com adição de albumina	Não

### Soluções e Insumos

As soluções utilizadas nas terapias extracorpóreas permitem a depuração direta das toxinas urêmicas, proporcionalmente ao fluxo de sangue na modalidade convencional, e em relação direta ao fluxo de dialisato nas terapias contínuas. A depuração de eletrólitos dependerá do gradiente entre a concentração sérica e a solução de dialisato, podendo ser ajustada conforme as concentrações dos eletrólitos em questão^66^.

Na terapia convencional, o dialisato é gerado em tempo real. Já na terapia contínua, poderão ser utilizadas soluções prontas, comercializadas, ou soluções preparadas na própria instituição, preferencialmente pela farmácia hospitalar. Quando a opção for o preparo pela equipe de enfermagem à beira leito, está indicado protocolo de segurança.

Essas soluções, juntamente com as soluções de citrato e de cálcio utilizadas na modalidade contínua, assim como as soluções empregadas na DP, deverão ser armazenadas em locais apropriados para sua adequada conservação.

### Acesso Vascular

A maioria dos pacientes hospitalizados com quadro de IRA e que necessitam de SRA deverá ter um cateter venoso temporário inserido. Eventualmente, para os pacientes estabilizados, nos quais se vislumbra intervalo de diálise mais prolongado ou definitivo, poderá ser indicado o implante de cateter de longa permanência.

A punção de veia central deve ser orientada por ultrassom, e a posição do cateter, após sua instalação, deve ser conferida radiologicamente^67^.

Os cateteres geralmente possuem diâmetro (French) de 11 a 14, havendo preferência para os de French 14 na modalidade contínua, particularmente quando há tendência à coagulação.

De acordo com o KDIGO, os cateteres devem ser implantados obedecendo a uma sequência preferencial de sítio de punção: veia jugular interna direita (16-20 cm), veia femoral – direita ou esquerda – (24-30 cm) e veia jugular esquerda (20-24 cm). A veia subclávia deve ser utilizada apenas em situações de exceção. O acesso por FAV fica reservado aos pacientes que se encontram em programa dialítico crônico e que permaneçam na modalidade convencional^67^.

### Monitoramento da Terapia

O monitoramento das TSR em pacientes hospitalizados, particularmente na modalidade de HD convencional, recai na observação da potencial instabilidade hemodinâmica, especialmente em pacientes com desnutrição, cardiopatias, em uso de drogas hipotensoras ou vasoativas (em doses baixas) e naqueles em transição da modalidade contínua^68,69^.

Recomenda-se que pacientes potencialmente instáveis sejam dialisados em ambiente de cuidados críticos e devidamente monitorizados, a fim de evitar episódios de hipotensão.

A monitorização hemodinâmica à beira leito, bem como a avaliação da volemia por meio de biomarcadores, bioimpedância, ecocardiograma e ultrassom *point of care*, permitem taxas de remoção mais acuradas e seguras^70,71^.

O controle bioquímico permite o acompanhamento da regularização de escórias pelas terapias e os consequentes ajustes nas doses de efluente. Além disso, o acompanhamento dos eletrólitos e do perfil ácido básico deve ser realizado, com as devidas correções feitas pelo perfil do dialisato, de acordo com a modalidade utilizada.

Na modalidade contínua, as análises bioquímicas são coletadas periodicamente, de duas a quatro vezes ao dia. Nessas coletas, também estão inclusas as análises relacionadas ao processo de anticoagulação utilizado, que, na maioria das vezes, é regional, utilizando-se o citrato trissódico. Nesse caso, o controle é feito pela determinação do cálcio iônico sistêmico e do circuito pós filtro^72^.

### Complicações

As complicações dos métodos de SRA estão relacionadas ao acesso vascular, ao circuito extracorpóreo ou ao próprio paciente.

Durante a inserção dos cateteres, podem ocorrer sangramento, hematoma, falha de obtenção de acesso ou não funcionamento primário, punção arterial, pneumotórax, hemotórax, embolia aérea e arritmias cardíacas. O médico que instala um acesso venoso deve estar ciente dessas possíveis complicações e ter condições de diagnosticá-las e manejá-las. Deve também avaliar previamente o paciente, reconhecendo coagulopatias, histórico de punções anteriores e a situação clínica atual. O uso do ultrassom para guiar a punção é uma medida preventiva indispensável^67^.

Após a inserção dos cateteres, podem ocorrer insuficiência de fluxo arterial ou venoso, desconexões acidentais, estenoses venosas, tromboses, infecções da corrente sanguínea ou do óstio. A prevenção dessas complicações passa por protocolos rigorosos de assepsia tanto na instalação quanto no cuidado dos cateteres. Além disso, deve- se evitar o uso do cateter para outras funções. A inserção do cateter adequado no vaso correto previne problemas posteriores.

As complicações relacionadas ao circuito extracorpóreo incluem embolia aérea, coagulação do sistema, hipotermia, falhas do equipamento e ruptura das fibras do dialisador. Os protocolos de anticoagulação e de manutenção dos equipamentos devem ser seguidos à risca. Devem ser evitadas taxas de fração de filtração superiores a 25%, assim como o desligamento dos alarmes do equipamento. É obrigatória, ainda, a monitorização contínua das pressões do circuito e de outros parâmetros de segurança.

Dentre as complicações relacionadas ao paciente, aquelas decorrentes da anticoagulação incluem sangramentos e trombocitopenia pelo uso de heparina, além de distúrbios eletrolíticos e ácido básicos, pelo uso do citrato.

A anticoagulação regional com citrato pode ocasionar: a) metabolização elevada ou falha na depuração do citrato, levando à sobrecarga, caracterizada por alcalose metabólica e hipernatremia, que podem ser corrigidas por meio de redução da dose, diminuição do fluxo de sangue, aumento na dose de diálise ou alteração na composição do dialisante ou reposição; b) intoxicação por metabolização inadequada (insuficiência hepática grave associada, hipoperfusão tecidual por choque refratário), caracterizada por redução do cálcio iônico sistêmico, aumento na relação cálcio total/cálcio iônico (> 2,5) – com o cuidado de verificar que ambos estejam expressos na mesma unidade de medida (mg/dL ou mmol/L) – e acidose metabólica com ânion gap elevado. Nesta última situação, a infusão do citrato deve ser interrompida.

O uso prolongado de altas doses nos métodos contínuos pode levar a distúrbios eletrolíticos, com depleção de íons intracelulares, hipofosfatemia, hipomagnesemia ou hipercalcemia secundária à reabsorção de cálcio ósseo por imobilidade prolongada. As soluções padronizadas, contendo concentrações fixas de eletrólitos e bicarbonato, podem tornar mais complexo o ajuste dos eletrólitos, equilíbrio ácido-básico e da anticoagulação.

Distúrbios eletrolíticos também podem ser induzidos na modalidade intermitente: variações rápidas nas concentrações de potássio, sódio e no equilíbrio ácido-base – o que deve ser previsto no planejamento do procedimento.

A instabilidade hemodinâmica é uma complicação frequente na condução do SRA. A hipotensão ocorre mais frequentemente nos métodos intermitentes, embora possa também ocorrer, eventualmente, nos métodos contínuos. A remoção rápida de líquidos, além da capacidade de reenchimento do compartimento intravascular, é a causa mais comum de instabilidade. Outras causas contributórias incluem desequilíbrios osmóticos, atordoamento do miocárdio, vasodilatação e aumento da permeabilidade capilar induzidas pela ativação imunológica no sistema extracorpóreo, sangramentos não aparentes, anemia, reativação de quadro séptico, isquemia mesentérica, acidose respiratória e depuração excessiva de vasopressores^73^. A avaliação frequente da hemodinâmica do paciente e da ultrafiltração efetiva obtida pode impedir uma remoção exagerada ou insuficiente de líquidos. A remoção excessiva pode comprometer a hemodinâmica do paciente e provocar aumento desnecessário na dose de drogas, hipoperfusão tecidual (principalmente em pacientes vasculopatas e idosos) e hiperlactatemia. Da mesma forma, o início tardio do período de ressuscitação, pode prolongar o tempo de recuperação, o tempo de suporte ventilatório, a síndrome compartimental, além do tempo excessivo e desnecessário de SRA.

Outras complicações relacionadas ao SRA incluem a remoção de nutrientes (aminoácidos, minerais, vitaminas, carboidratos, elementos essenciais – como selênio) e de drogas. A nutrição do paciente deve ser ajustada de acordo. Em alguns casos, pode ocorrer cetoacidose euglicêmica^74^.

A farmacocinética de diversas drogas, especialmente antibióticos, é alterada pelos métodos de SRA. Métodos contínuos removem mais eficientemente vários antibióticos, que devem ser administrados em doses habituais, não reduzidas pela função renal (meropenem, vancomicina). Sempre que possível, os níveis séricos devem ser monitorados. Já os métodos intermitentes requerem a administração de doses adicionais após o procedimento. Esses aspectos terapêuticos devem ser de conhecimento do nefrologista, que deve orientar a equipe assistente a respeito. Como a Monitorização Terapêutica das Drogas não está disponível para a maioria dos antibióticos, os ajustes de dose devem ser feitos com base na dose de terapia, método e frequência do SRA. Algumas tabelas de correção publicadas recentemente podem ser consultadas^75^.

### Qualidade e Segurança

Recomenda-se que os serviços de Nefrologia que prestam suporte dialítico avaliem rotineiramente a qualidade dos serviços realizados. Vários parâmetros podem ser utilizados, como o tempo entre a indicação e o início da terapia, o consumo de insumos (filtros, soluções e cateteres), a dose de terapia e de ultrafiltração efetiva, o tempo de uso dos equipamentos, o número de complicações relacionadas ao tratamento e a capacidade de resolução^76,77^.

Também devem ser monitorados desfechos principais, como mortalidade, tempo de hospitalização, tempo de permanência em UTI, recuperação da função renal e encaminhamento para terapia dialítica crônica^76,77^.

Recomenda-se que as normas recentes da ANVISA, padronizadas para os serviços de hemodiálise e que regem as práticas de segurança para os pacientes, sejam seguidas para os serviços que prestam SRA hospitalar. Nesse sentido, deverão ser avaliados mensalmente os seguintes indicadores: plano de segurança do paciente; higiene das mãos; identificação do paciente; prevenção de quedas; protocolo de segurança na prescrição e administração de medicamentos; prevenção de eventos adversos relacionados ao acesso vascular; prevenção de infecções e eventos adversos em diálise peritoneal; prevenção de coagulação do sistema durante a terapia extracorpórea; prevenção e controle de transmissão de microrganismos resistentes, bem como de HIV e hepatites B e C; protocolo implantado de monitoramento da qualidade da água de hemodiálise; gerenciamento de tecnologias (equipamentos); e notificação de incidentes relacionados aos colaboradores^78,79^.

A avaliação da qualidade da água e o controle de infecções relacionadas à assistência nefrológica são obrigatórios para os serviços de Nefrologia em âmbito hospitalar e serão descritos a seguir^54,58,78,79^.

### Controle da Água

Os parâmetros para a coleta e análise microbiológica da água potável e tratada, bem como os procedimentos de manutenção do sistema de armazenamento, devem seguir as determinações previstas na RDC nº 11, de 13.03.2014, da ANVISA^58,78^.

O cronograma para a coleta e análise microbiológica da água potável e tratada deve ser estabelecido pelo Serviço de Controle de Infecção Hospitalar (SCIH).

O hospital deve dispor de registros diários e mensais das análises das características físicas, organolépticas e microbiológicas da água potável disponibilizada para a terapia dialítica.

O serviço de Nefrologia deverá manter registros mensais e semestrais das análises da água tratada, com suas características microbiológicas e físico-químicas, bem como o calendário de desinfecção e troca das membranas e filtros dos equipamentos.

### Controle de Infecções

Com o objetivo de reduzir as infecções relacionadas à assistência ao paciente dialítico, os hospitais devem, por meio do SCIH, disponibilizar capacitação profissional e registros específicos com foco em: limpeza e desinfecção de equipamentos; cuidados na manipulação de cateteres de hemodiálise e diálise peritoneal, bem como rotinas de inserção e manutenção; medidas adotadas durante episódios de bacteremia nas sessões de diálise; orientações sobre limpeza de artigos, superfícies e descarte de resíduos e materiais; ações a serem tomadas diante de laudos de qualidade da água em desacordo com os padrões exigidos; orientações sobre o uso de EPIs pelos profissionais; orientações sobre os processos a serem adotados para pacientes colonizados por bactérias MR ou portadores de doenças respiratórias transmissíveis^58,77,78,79^.

### Registros

Os registros dos atendimentos realizados pela equipe multiprofissional em Nefrologia devem ser feitos em prontuários padrão do hospital. Devem constar no registro médico: história clínica, avaliação diária, justificativa para início de terapia dialítica, escolha da modalidade, métodos e tipo de acesso. Além disso, devem ser incluídas as avaliações médicas realizadas antes do início dos procedimentos, bem como as orientações referentes às mudanças de parâmetros durante as terapias.

As prescrições médicas devem ser realizadas de forma detalhada antes do início de cada terapia, e quaisquer alterações necessárias devem ser registradas em prontuário ou documento padronizado, com assinatura e carimbo do profissional responsável.

Os registros da enfermagem e dos técnicos, nos prontuários, devem incluir as avaliações de rotina, a descrição operacional do início, manutenção e término das terapias, bem como o relato de eventos e intercorrências.

Aspectos relacionados aos acessos, à manipulação e à manutenção também devem ser devidamente registrados.

## Seguimento Ambulatorial de Pacientes Com Injúria/Doença Renal Aguda

Pacientes com IRA, especialmente aqueles internados em UTI, apresentam alta morbimortalidade nos meses subsequentes à alta hospitalar. Há risco elevado de complicações clínicas e reinternações, particularmente em pacientes portadores de diabetes, doença cardiovascular e DRC^80-82^.

Os pacientes com IRA podem evoluir das seguintes formas:

a. Resolução imediata antes de 7 dias;
b. Resolução imediata, com recaídas antes de 90 dias;
c. Resolução tardia em até 90 dias;
d. Sem resolução em até 90 dias, com evolução para DRC.

Denomina-se Doença Renal Aguda (DRA) a persistência da disfunção/lesão renal aguda ou subaguda, manifestada por estágio IRA KDIGO ≥ 1, entre 7 e 90 dias após um quadro de IRA^83,84^.

Pacientes portadores de IRA, mesmo em estágios iniciais, apresentam maior risco de complicações cardiovasculares, óbito e desenvolvimento de DRC^84^.

Desta forma, recomenda-se aos pacientes sobreviventes de IRA, após a alta hospitalar:

1. Seguimento ambulatorial por médico assistente, com avaliação da função renal: dosagem de creatinina sérica e albuminúria, em prazo não superior a 3 meses após a alta;
2. Quando houver persistência da elevação da creatinina e/ou albuminúria após 3 meses do quadro de ira, estes pacientes devem ser acompanhados por nefrologistas, conforme as diretrizes para drc;
3. Nos pacientes com recuperação completa da ira, recomenda-se avaliação renal periódica (dosagem de creatinina sérica e albuminúria), pelo menos após um ano da alta hospitalar;
4. Pacientes com ira persistente, portadores de outras nefropatias e drc, devem ser encaminhados ao nefrologista para seguimento ambulatorial.

## Data Availability

Todo o material bibliográfico utilizado na presente guia, encontra-se disponível com os autores.
